# Local knowledge and socio-economic determinants of traditional medicines' utilization in livestock health management in Southwest Nigeria

**DOI:** 10.1186/1746-4269-8-2

**Published:** 2012-01-12

**Authors:** Taiwo E Mafimisebi, Adegboyega E Oguntade, Adebowale N Fajemisin, Olaiya P Aiyelari

**Affiliations:** 1 Department of Agricultural Economics and Extension, The Federal University of Technology, Akure, Nigeria; 2 Department of Animal Production and Health, The Federal University of Technology, Akure, Nigeria; 3 Department of Crop, Soil and Pest Management, The Federal University of Technology, Akure, Nigeria

**Keywords:** Medicinal Plants, Livestock, Health Management, Local Knowledge, Determinants, Utilization, Nigeria

## Abstract

Smallholder livestock farmers in Nigeria utilize traditional medicines derived from medicinal plants (PMs) for the maintenance of their animals' health. This study was designed to determine the PMs used in the study area and their level of utilization by livestock farmers, compare the level of utilization of PMs across the three states surveyed and identify the socio-economic factors influencing farmer's utilization of PMs. Thirty-five PMs were identified. Farmers had considerable knowledge about the identified PMs but about 80.0% of them used the PMs to poor/moderate extent. There were statistical differences in the utilization level of PMs among the three states. Six socio-economic variables were found to be statistically significant in influencing PMs' utilization. Farmer's age, household size, distance to the nearest veterinary hospital/clinic and extent of travels, had positive effects while negative effects were exhibited by farm income and number of heads of livestock. It was concluded that there was considerable knowledge about PMs and that utilization of PMs varied between the three states. It was recommended that local knowledge of PMs be preserved in the study area through screening and documentation.

## Introduction

The Nigerian livestock industry is performing below expectation in its role of providing adequate animal proteins for the growing population. While annual population growth rate is about 3.0%, the estimated annual growth rate of the outputs of the major livestock products is nearly stagnant. Also, the contribution of the livestock sub-sector to the Gross Domestic Product (GDP) has been declining [1-3]. This poor performance of the Nigerian livestock industry is in spite of an estimated livestock population of about 600 million [1]. The per *capita *animal protein intake is below 7.0 g per day [4-7]. With reference to the 35.0 g of animal protein per *caput *per day recommended by FAO, the shortfall in minimum protein requirements is about 89.0% [7,8]. In fact, by the year 2020 when the Nigerian population is expected to be about 230 million, a quantum leap in livestock production is required if the seemingly formidable problem of protein malnutrition is to be surmounted.

It is a general consensus that the generic problem of low productivity of the existing livestock population is the factor responsible for Nigeria meeting just about 50.0% of her per *capita *demand for animal proteins from domestic sources [6]. Associated with this generic problem are specific constraints which include the traditional animal rearing system characterized by low productivity, high mortality rate, low growth rate, prevalence of pests and diseases and other factors which render efficient use of resources impossible [2,6].

The problems of pests and diseases and high mortality rates necessitate that health issues in traditional livestock management be tackled decisively for a successful livestock development programme in Nigeria. This is necessary because researchers have found out that healthcare expenses constitute close to 20% of the production cost of livestock in Nigeria [2,7]. Since the massive devaluation of the Nigerian naira in June, 1986, the currency has been losing value leading to increases in the prices of agricultural inputs, especially veterinary drugs, which are usually imported. The consequence of this is that poor livestock farmers were priced out of the market for imported inputs [6]. In three out of the five ecological zones in Nigeria, all (100%) of the sampled farmers rated high cost of veterinary drugs as an important constraint to livestock production [9]. The high prices of inputs are the main cause of the collapse of many commercial livestock concerns. Smallholder livestock farmers were then left with the option of using more of PMs in managing the health of their animals to that livestock production can remain competitive [10].

According to McCorkle [11,12], "the fact remains incontrovertible that from time immemorial, traditional stock raisers; farmers and herders, have developed and perfected their own ways of managing the health of their stock thereby keeping them productive". Traditional livestock keepers were reported to treat and prevent livestock diseases using age-long home remedies, surgical and manipulative techniques, husbandry strategies and magico-religious practices [12]. The totality of these indigenous local animal healthcare beliefs and practices is what is popularly referred to in the literature as ethno-veterinary medicine [11].

Nigeria, like any other typical tropical country, is a paradise of parasites [13]. Though the nation's livestock population seems numerous, the health hazards and high mortality rates due to various diseases and parasitic infestations have been a vital and serious threat to livestock development [2]. About 95% of the livestock in Nigeria are owned by the rural farmers. These farmers are mostly without western education and generally trapped in poverty. Most of them cannot appreciate the importance of modern veterinary healthcare in livestock management. Where they do, they probably cannot afford the prohibitive costs of the services some of which are being provided by quack veterinary personnel. This is probably responsible for the higher incidence of pests and diseases and mortality rates in the livestock managed by rural compared with peri-urban and urban farmers [8,14-16].

There are remarkable drawbacks of traditional medicines emanating from locally available medicinal plants (henceforth PMs) in terms of their preparation, efficacy, disease diagnosis and treatment specifications [17]. Despite this, their potential in the management and rearing of livestock in low-income developing countries cannot be denied [17]. According to McCorkle [12], the effective remedies and practices have several areas of high point compared with western drugs. They are cheaper because the important raw materials (plants) are obtained at no cost. In addition, they are more accessible and more readily understood. They also have a higher degree of environmental friendliness and are often more socio-culturally acceptable and better adapted to local realities.

A medicinal plant is any plant, which in one or more of its organs, contains substances that can be used for therapeutic purposes or which are precursors for the synthesis of useful drugs [18]. According to Ogunlela [19], ''numerous plant species have several medicinal uses and perhaps, there is hardly any plant known to man that has no use in medicine." Ogunlela [19] also argued that most of the drugs that feature in pharmaceutical industry derive their origin from one or more plant products with different plant parts having different uses in the treatment of human and animal ailments. Medicinal plants are then best described as the bedrock of the pharmaceutical industry. This is probably the reason why medicinal plants are assuming increasing importance and relevance in various communities, especially in developing countries such as Nigeria. It is in these parts of the world that the curative effects of the various parts of such plants are most appreciated [19]. In rural areas of developing countries, probably the only avenue for healthcare delivery and treatment of sick humans and livestock is the practice of traditional medicines derived mostly from medicinal plants [20].

The extent of knowledge and utilization of PMs differ according to the culture and prevailing socio-economic conditions of users. Thus, it is important to provide empirical answers to questions like when and why do farmers use traditional medicines in the management of their stock health. Worldwide, adoption of improved livestock health management practices and the relationship between the adoption behaviour of farmers and farmers' characteristics have been studied [7,21-25]. In Nigeria, however, the actual extent of use of traditional medicines derived from medicinal plants as well as factors responsible for differences in their utilization among farmers in same and/or different localities have been sparingly explored. A careful investigation into this will contribute to improving Nigeria's livestock health management policies. This will help ensure the sustainability of livestock production and guarantee increased supply of affordable animal proteins. This study is therefore directed at (i) identifying PMs being used by farmers in livestock health management, (ii) determining the extent to which the identified PMs are being utilized by farmers, (iii) comparing the extent of use of PMs between farmers in identified locations of the study area, and (iv) investigating the relationship, if any, between farmers' socio-economic characteristics and their utilization of PMs.

## Methods

### Study Area, Sampling and Data Collection

The study was carried out in Southwest Nigeria. Nigeria lies on 10° 0' 0" N latitude and 8° 0' 0" E longitude. Farming is a major source of livelihood to the people of the Southwest Nigeria [26]. Traditional livestock rearing, especially small ruminants and poultry, is an important agricultural enterprise in the region. Data were gathered by administering a structured questionnaire on livestock farmers in one purposively selected Local Government Area (LGA) in three (Ondo, Ogun and Oyo) out of the six states in the region. The selection of these states was based on the fact that they had the highest number of registered farmers in Southwest Nigeria. Okitipupa LGA was selected in Ondo State, Iddo LGA was selected in Oyo State while Odeda LGA was selected in Ogun State. The LGAs were purposively selected based on the fact that they were previously hosts to farm settlement schemes. The farm settlement schemes were government sponsored agricultural programmes aimed at attracting young and educated persons into commercial agriculture. Most of them were established in the late 1960s. The existence of farm settlement schemes in these LGAs is expected to have exposed the farmers to veterinary drugs for animal health management and, hence, the farmers should be able to compare them with traditional medicine for managing animal health. Based on information supplied by the Agricultural Development Project (ADP) office of each state on the number of registered livestock farmers, three (3) communities with the highest number of registered livestock farmers were selected from each LGA. From each community, twenty (20) farmers were selected for interview by simple random sampling. Only farmers rearing small and large ruminants were targeted for the study. Data were gathered from the sample farmers using questionnaire.

The development of the interview schedule took place in two phases. In the first phase, an interview schedule with open-ended questions was used to identify the most viable PMs being used in the study area through extensive field visits, key informant interviews and focus group discussions (FGDs). The identified PMs were listed in a structured interview schedule for final administration.

A total of 180 farmers were selected for interview. However only 120 set of questionnaire contained adequate information for analysis. The set of 120 questionnaire is distributed as follows: Okitipupa LGA, 38; Iddo LGA, 34 and Odeda LGA, 48.

### Data Analysis

The analytical tools used included descriptive statistics such as frequency tables, percentages, inferential statistics and regression model. The extent of use of PMs was expressed by the use of a five-point rating scale with the scoring order of 5,4,3,2 and 1 for frequently used (FU), occasionally used (OU), rarely used (RU), aware but not used (ANU) and not aware (NA), respectively. Two methods of computation were employed in capturing the utilization of PMs. The first one is the PM Use Index (PMUI). Following from Islam and Kashem [27], the PMUI for each PM was calculated as follows.

(1) $$\text{PMUI = }{\text{N}}_{1}\times 5+{\text{N}}_{2}\times 4+{\text{N}}_{3}\times 3+{\text{N}}_{4}\times 2+{\text{N}}_{5}\times 1$$

Where

PMUI is as already defined

N_1 _= No of farmers using PMs frequently

N_2 _= No of farmers using PMs occasionally

N_3 _= No of farmers using PMs rarely

N_4 _= No of farmers aware of but not using PMs

N_5 _= No of farmers not aware of PMs

The PMUIs were also used to rank the PMs in terms of their use.

The second represents the use of PMs by individual farmers. This level of utilization of PMs for each respondent farmer was calculated by adding up the scores obtained by the farmer in all the identified PMs compiled for the study. The scores obtained by the farmers were categorized into low user (55-90), moderate user (91-126) and high user (127-162) and presented in a frequency distribution.

The z-test was used to examine whether or not there was significant difference in the level of use of PMs between farmers in a pair of states among the three states covered in the survey. The formula is as presented below in comparing the level of use of PM for farmers for any combination of two of the three states.

(2) $$z=\frac{{\bar{X}}_{1}-{\bar{X}}_{2}}{\sqrt{S_{1}^{2}/n_{1}}+\sqrt{S_{2}^{2}/n_{2}}}$$

Where z = standard "Z" distribution value (z calculated)

$\overset{-}{X_{1}}$= mean value of PM level of use for state (1) farmers

$\overset{-}{X_{2}}$ = mean value of PM level of use for state (2) farmers

S_1 _= standard deviation of sample mean value of PM level of use for state (1) farmers

S_2 _= standard deviation of sample mean value of PM level of use for state (2) farmers

(1) and (2) = any two combination of Ondo, Ogun and Oyo States

n_1 _= sample size for Ogun State (48)

n_2 _= sample size for Ondo State (38)

n_3 _= sample size for Oyo State (34)

Ordinary least squares regression was used in identifying farmers' socio-economic characteristics which influence the use of PMs. The implicit form of the regression equation is:

Y = f (X_1_, X_2_, X_3_, X_4_, X_5_, X_6_, X_7_, X_8_, X_9_, X_10_, U_t_)

Where Y = level of use of PMs by farmers

X_1 _= average yearly income from farming activities (N)

X_2 _= farmers' age (years)

X_3 _= farmers' number of years of formal education

X_4 _= farming experience of farmer

X_5 _= household size

X_6 _= number of heads of animals kept

X_7 _= average number of travels by farmers outside farmers' own community per year

X_8 _= membership of trade/co-operative associations, dummy, 0 if farmer is not a member, 1 otherwise

X_9 _= mass media exposure of the farmer as indicated by number of information media being accessed

X_I0 _= distance of farmers' farm to the nearest veterinary clinic/hospital (km)

U_t _= Error term assumed to be stochastic.

There was no assumption as to the regression form which gives the best fit. Four functional forms; linear, exponential, double-log and semi-log were each fitted to the data. The lead equation was selected based on statistical, economic and econometric criteria.

## Results and Discussion

### Background Information

Empirical result showed that about 50.0% of the farmers have been into livestock rearing for ten years or less. Close to 36.0% of the farmers had experience of between 11-20 years while the balance of 15.0% had been in the animal rearing and production venture for 20 years and over (Table 1). About 67.0% of the farmers had less than secondary school education. The farmers that had secondary and tertiary education were about 33.0% of the respondents. The poor level of education is a reflection of the situation obtainable in the rural areas of Nigeria [26]. About 87.5% of the respondents were male. This is probably due to the fact that household heads in Nigeria are predominantly male [26].

**Table 1:** Socio-economic Characteristics of Livestock Farmers

Farming experience (yrs)	No of farmers	Percentage	Cumulative percentage
1-10	59	49.2	-

11-20	43	35.8	85.0

21-30	18	15.0	100.0

**Educational level**			

No formal education	26	21.7	-

Adult literacy education	18	15.0	36.7

Primary school	36	30.0	66.7

Secondary school	30	25.0	91.7

Tertiary school	10	8.3	100.0

**Sex**			

Male	105	87.5	87.5

Female	15	12.5	100.0

Types of ruminants*			

Goat	96	80.0	-

Sheep	61	50.8	-

Cattle	26	21.7	-

*Most farmers kept more than one type of livestock.

The livestock most frequently kept were goats and sheep. Goats were being kept by 80.0% of the sample farmers while about 51.0% of the farmers were keeping sheep. Rearing of cattle was rare especially in Ondo and Ogun States while in Oyo State; a few farmers were having cattle as part of their stock. In all, only 16.0% of sample farmers had cattle as part of the livestock being reared (Table 1).

In terms of how knowledge of PMs was transferred, 40% of the sample attributed it to their parents while 27% traced it to other farmers and friends involved in livestock rearing both within and outside their communities. Close to 14% of the respondents said they sometime receive information on medicinal uses of herbs, their preparation and administration from dreams. It is their belief that farmer's departed forefathers and guiding spirits of the community sometime reveal such to a helpless person to save human or animal live. Folkloric tales and trial and errors (local experimentation which leads to experience over years) account for 15% while the balance of 4% is due to informal training. Most of the farmers interviewed were of the opinion that traditional knowledge of uses of medicinal plants is currently under threat with the imminent exit of the older generations, who had the custody of traditional medical practice. This position is in accordance with the observation by Prance [28], that these older people have the bulk of the information on uses of plants for medicine. This, he said, calls for better focus on conserving traditional community knowledge of remedies and scientific evaluation of safety and efficacy PMs.

### Level of Knowledge and Utilization of PMs

Thirty-five (35) PMs identified in the study area are shown in Table 2. The PMs listed in the table are unique medical practices based on ethno-botanical resources available in the rural areas. The necessary ingredients are locally available, free or are very cheap and are thus expected to fit the culture and the socio-economic realities of farmers especially those that are poor. These PMs are thus potential valuable tool(s) in the design of sustainable health component of traditional animal husbandry strategies for the smallholders of Southwest Nigeria. Most of the plant sources presented in Table 2 have been studied and their active ingredients are scientifically associated with the effects that farmers claim they produce both in Africa and other countries [18,27].

**Table 2:** Plant Medicines Identified Based on Plant Medicines Use Index

No.	Plant Medicine Practice	PMs Scores					PMUI
			
		FU	OU	RU	ANU	NA	
1	Deworming of livestock through extract of tender shoots of pineapple plant (*Ananas comosus*).	42		10	10	8	468

2	Ground garlic and ginger mixed with palm oil for curing cough.	44	36	10	26	4	450

3	Feeding the juice of old sugarcane (last season's crop) as a cure for cessation of urination.	40	38	20	12	10	446

4	Treatment of sleeplessness with extract from goat weed (*Ageratum conyzoides*) leaves sprinkled with chewed alligator pepper.	40	48	8	8	8	440

5	Feeding raw ginger mixed with table salt to cure constipation.	40	30	26	8	16	430

6	Control of body lice through application of leaf extract of bullock's heart (*Annona recticulata*).	32	40	26	6	16	426

7	Use of root and stem extract of snakewort in stimulating uterine contraction and parturition in pregnant animals.	40	21	30	18	11	421

8	Use of snakewort (*Aristolocia bracteata*) leaf extract as antidote to snake poison, scorpion bite and antihelmintic.	29	40	20	20	11	416

9	Use of extract of cashew tender roots in curing persistent cough.	30	40	21	10	19	412

10	Use of *Euphorbia hirta *leaves in the treatment of diarrhoea and dysentery.	26	36	28	20	10	408

11	Treatment of physical weakness by feeding raw ginger and molasses paste to an animal once daily.	40	35	12	20	13	394

12	Use of *Euphorbia hirta *leaves pounded and mixed with water as cure for constipation.	30	22	29	20	19	384

13	Feeding gur (juice of sugarcane in crystallized form) to cure cessation of urination.	29	16	36	24	15	380

14	Feeding of gum of tamarind and banana together with straw to improve lactation on first delivery.	21	37	19	26	17	379

15	Application of extract of wide cucumber (*Mormodica charalia*) as purgative and antidote to some gastro-intestinal disorders.	24	26	31	22	17	378

16	Use of dog's liver plant (*Kelanchoe crenata*) leaf extract in treating general debility and stiffness of joints.	18	31	36	20	15	377

17	An infusion of hairy spurge or Australian asthma herb (*Euphorbia hirta) *given to animals for increased lactation.	17	38	25	24	16	376

18	Use of the root and bark of Radish plant (*Moringa pterygosperma*) in treatment of fever and mouth sores.	17	31	34	26	12	375

19	Application of ground seeds of wild cucumber in treatment of jaundice.	26	19	28	37	10	374

20	Cultivation of *Ocimum gratissimum *and *Cymbopogon citrates *round livestock houses to serve as repellents to insects.	26	31	20	14	29	371

21	Crushed back of Wild Oliver applied to sores of domestic animals.	20	26	32	19	23	361

22	Use of *Ocimum gratissimum *leaf and whole plant part in treatment of diarrhoea. The animal drinks the cold infusion extract twice daily.	16	20	40	36	8	360

23	An infusion of crushed fruit of wild Oliver given to animals to cure "bloody diarrhoea".	18	24	30	32	16	356

24	Curing loose motion through feeding of leaves of *Musa acuminata *mixed with the ash of dried walnut.	12	28	41	20	19	354

25	Use of *Ageratum conyzoides *in dressing sores and wounds.	18	27	29	18	28	349

26	Use of mustard oil paste to cure gingivitis.	16	24	32	28	20	348

27	Using a paste of lime juice, butter and few seeds (10-12) of black pepper to cure asthma/anorexia.	16	28	14	32	29	339

28	Curing digestive troubles through feeding of rashun, lime juice and butter wrapped in banana leaves	11	28	40	10	31	338

29	Applying few drops of juice of immature *Leucus aspera *mixed with table salt at the corner of the eyes to cure cessation of defecation	14	26	30	22	28	336

30	Extract of roots of wild Oliver (*Ximenia americana*) with that of *Annona senegalensis *or *Annona. chrysophylla *in the treatment of sleeping sickness.	10	28	31	29	22	335

31	Coating the area of pain with molasses and ash of *Harrisonia abyssiniea *to cure haemorrhagic septicemia.	22	12	30	30	26	334

32	Pouring of raw mustard oil through the nostril of an animal to stimulate appetite.	18	22	31	12	37	332

33	Curing bloat through dilution of dusts of turmeric in indigenous smoking pipe and feeding it to the animal.	14	26	24	21	32	320

34	Application of *Solanum nodiflorum *wrapped in banana leaves and heated in hot ash. The juice is squeezed on to the sore and applied twice daily.	26	30	31	23	10	299

35	Use of aqeous leaf extract of *Hagenia abyssinica *and *Mollugo hirta *as anthelmintic in vitro.	16	14	24	20	46	294

NB: FU = frequently used, OU = occasionally used, RU = rarely used, ANU = aware but not used, NA = Not aware

Table 3 presents information on the level of knowledge of PMs among the sampled farmers. The table shows that the sum total of farmers who claimed to know the remedies presented in Table 2 (summation of frequently used (FU), occasionally used (OU), rarely used (RU) and aware but not used (ANU)) was 3,549 while the sum total of farmers who claimed not to know the remedies (summation of not aware (NA)) was 651. The mean number of farmers who claimed to know the 35 remedies in Table 2 (3,549 divided by 35) was 101.4 while the mean number of farmers who were not aware of the remedies (651 divided by 35) was 18.6. These mean values indicate that about 101 farmers out of 120 acknowledged that they know the remedies. This represents about 84.5% of the sampled farmers.

**Table 3:** Farmers' Knowledge of the Thirty-five Plant Medicines

	Had Knowledge	Lacked Knowledge*
Total	3549	651

Mean	101.4	18.6

Percent	84.5	15.5

* Computed from NA (Not Aware) column of Table 2

Going by PMUIs, the PMs contained in Table 2 were categorized as poorly-used, moderately-used and highly-used. The PMUIs ranged between 294 and 468. On a fairly equal interval scale, about 31.0% of the PMs were poorly-used while 43.0% and 26.0% were regarded as moderately- and highly-used, respectively (Table 4). These findings are similar to those of Islam and Kashem [27]. The fact that none of the PMs had a PMUI of zero indicates that PMs have not been completely discarded by farmers in spite of the availability of western veterinary drugs. This may mean that they probably benefit the farmers. The PMs that are poorly used by farmers may have been so used because they were known to a restricted number of farmers in the locality. It is also possible that certain PMs were avoided by farmers because of repeated poor efficacy or higher complexity in both preparation and use. Also, poor use of a PM may arise from the availability of modern means of treatment that are more convenient than the corresponding PM [27].

**Table 4:** Classification of Plant Medicines based on Plant Medicines Use Index

Categories based on Plant Medicines Use Index	Number of Plant Medicines in each category	Percentage
Poorly-used (294-351)	11	37.4

Moderately-used (352-410)	15	42.9

Highly-used (411-468)	9	25.7

Total	35	100.0

The attributes of a new practice that may give rise to poor adoption are lower relative advantage, higher complexity and lower compatibility [29]. The PMs in the poorly-used category having lower PMUI and lower ranks are probably characterized by these afore-stated attributes and are probably on their ways to extinction in traditional livestock health management by farmers. On the other hand, the highly used PMs may be relatively advantageous, less complex and more compatible with the needs and realities of the farmers. Farmers may also have been utilizing these practices because of inaccessibility to the corresponding modern remedial measures [27].

Table 5 reveals that about 35.0% of farmers used the PMs moderately while only 20.0% used PMs highly. The remaining 45% of the farmers used PMs poorly. This finding attests to a significant knowledge and use of the PMs among rural livestock farmers in the study area. Also, no individual respondent recorded a zero score in the use of PMs. This implies that the PMs probably offer some positive benefits to users.

**Table 5:** Level of Utilization of Plant Medicines

Categories	Respondents	
	
	Number	Percent
Low user (55-90)	54	45.0

Moderate user (91-126)	42	35.0

High user (127-162)	24	20.0

Total	120	100.0

Mean = 66.849 Standard Deviation = 28.489

### Comparison of PM Level of Use between States

The mean PM level of use for Ogun, Ondo and Oyo States were 67, 56 and 59; respectively. The results of the pair-wise comparison by z-test (Table 6) showed that there was a significant difference in mean PM level of use between Ogun and Ondo States as was also the case between Ogun and Oyo States at the 5% level of significance. However, there was no significant difference in PM level of use between Ondo and Oyo States. The implication of these results is that individual farmer's extent of use of PM was higher in Ogun State compared with Ondo and Oyo States. A deliberate regional policy to encourage farmers' use of PMs in Southwest Nigeria should therefore commence in Ondo and Oyo States.

**Table 6:** Comparison of Mean Plant Medicines Level of Use across States

Locations	Mean Plant Medicines Level of Use			Z value	P value
	**Ogun**	**Ondo**	**Oyo**		

Ogun Versus Ondo	67 (18.4)	56 (22.5)	_	11.08	0.044

Ogun Versus Oyo	67 (18.4)	_	59 (26.2)	13.62	0.041

Ondo Versus Oyo		56 (22.5)	59 (26.2)	5.26	0.112

*Values in parentheses are standard errors

A further comparison of PMUI across the three states is presented in Table 7. The table shows that, among the high-users of PMs, Ogun State accounted for 66.7%, while Ondo and Oyo States accounted for 20.8% and 12.5%, respectively.

**Table 7:** Comparison of Level of Utilization of Plant Medicines

States	Category		Category		Category		Total
		
	Low Users		Moderate Users		High Users		
		
	No	%	No		No	%	
Ogun	13	24.1	19	45.2	16	66.7	48

Ondo	24	44.4	09	21.4	05	20.8	38

Oyo	17	31.5	14	33.3	03	12.5	34

Total	54	100.0	42	100.0	24	100.0	120

### Determinants of PMs' Utilization

Upon critical evaluation of the results of the various functional forms, the double-log form of the regression model was chosen as the lead equation. The results which reveal the level of variation in extent of use of PMs (Y) explained by farmer-specific independent (explanatory) variables is presented in Table 8.

**Table 8:** Multiple Regression Results

Variable	Coefficient	Standard Error	Level of significance
Constant	-101.41	726	

Average yearly income from farming activities	- 20.29	4.40	1%

Farmers' age (years)	14.35	6.33	5%

Farmers' number of years of formal education	- 6.45	12.21	Not significant

Farming experience of farmer	3.44	7.84	Not significant

Household size	10.55	4.26	5%

Number of animals kept	-19.21	5.11	1%

Average number of travels by farmers outside farmers' own community per year	11.21	4.92	5%

Membership of trade/co-operative associations	-7.24	6.81	Not significant

Mass media exposure of the farmer as indicated by number of information media being accessed	- 4.87	2.96	Not significant

Distance of farmers' farm to the nearest veterinary clinic/hospital (km)	12.52	4.77	5%

Dependent variable is the natural logarithms of the level of use of Plant Medicines. The independent variables are all in natural logarithms.

R^2 ^= 0.642; R^-2 ^= 0.629; F = 36.21

The model's R^2 ^means that the postulated socio-economic variables were jointly responsible for about 64.0% of the observed variations in the utilization of PMs. The results reveal a positive significant relationship between farmer's age, household size, distance of farmers' farm to the nearest veterinary clinic/hospital and the average number of times a farmer has travelled outside his/her locality in a year; and the utilization of PMs. These variables were significant at the 5% level. Higher values of these variables will translate to higher level of PMs' utilization.

The positive relationship between age and utilization of PMs can be explained from the perspective of older farmers being custodians of knowledge on the preparation and application of PMs [30]. Also, the longer years of acquaintance with PMs will enable older farmers to be sure of the efficacy or otherwise of some of the PMs and the conditions to be fulfilled in their preparation to enhance efficacy [30].

Travelling education broadens one's intellectual and information horizons through extended contact with outside knowledge and information sources [27]. A farmer that travels more often has a greater opportunity of meeting different persons or receiving information from sources unavailable in his/her community. This makes it possible for such a farmer to have knowledge of PMs and modern veterinary services available elsewhere. S/he is therefore able to compare and select a combination of PMs and modern veterinary practices that is more cost-effective. This may be the reason for the observed positive relationship between the average number of times a farmer has travelled outside his/her locality and utilization of PMs.

Household size increases brought about an increased utilization of PMs probably because the higher the household size, the higher the household expenditure. For a poor farmer, the competition for funds between household needs and livestock health maintenance may elicit the response that the farmer holds on to the cheaper PMs to free sufficient financial resources for meeting household expenditures. The regression results indicate that a 1% increase in each of farmer's age, household size and average number of times a farmer has travelled outside his/her locality will result in 14.4%, 10.6% and 11.2%, respectively in the level of use of PM. Similarly, a 1% increase in the distance of farmers' farm to the nearest veterinary clinic/hospital will result in 12.5% increase in the use of PM.

Variables that showed significant negative relationships with the utilization of PMs were farm income and heads of livestock kept. For these variables, the higher their values, the lower the utilization of PMs. From the results, a 1.0% increase in farm income translates to a 20.0% decrease in the utilization of PMs. Likewise; a 1.0% increase in the heads of livestock kept will result in 19.0% decrease in the utilization of PMs.

The negative relationship between farmer's income and utilization of PMs is explainable from the view point that a farmer's economic position will influence his access to modern veterinary services in rearing and managing livestock health. The higher the income, the higher is the access to veterinary drugs. Higher economic status may therefore reduce farmer's use of PMs [20,30]. Also, increase in the number of animals kept led to reduced utilization of PMs probably because the higher the number of animals, the higher the tendency to rear the livestock on commercial basis and hence the higher the use of purchased inputs which invariably include veterinary drugs.

## Conclusion and Recommendations

This study sets out to determine the PMs used in the study area and their level of utilization among livestock farmers. It was also intended to compare the level of utilization of PMs across the three states and identify the socio-economic factors influencing farmer's utilization of PMs.

A total of 35 PMs were identified in the study area. There was considerable knowledge about these PMs among the livestock farmers. In spite of this, most (80.0%) of the farmers used PMs to poor/moderate extent. The level of utilization of PMs varied between the three states, with Ogun State taking the lead. Six socio-economic variables influenced the utilization of PMs significantly. These are farmer's age, household size, distance of farmers' farm to the nearest veterinary clinic/hospital and extent of travels; which had positive effects; and farm income and heads of livestock; which had negative effects.

There was significant knowledge about PMs in the study area and, due to continuing operations of the farmers at subsistence level; PMs will most likely continue to be relevant for livestock health management. There is a need to preserve local knowledge of PMs and this could be achieved through Farmer Field Research in which university researchers and farmers jointly carry out research about PMs on farmers' farms. This will enable groups of farmers and researchers, through a discovery learning process, find out about the efficacies of various PMs while the younger farmers and university researchers learn from the older farmers who are regarded as custodians of knowledge about PMs. This process will facilitate the documentation of PMs with proven efficacies.

## Competing interests

The authors declare that they have no competing interests.

## Authors' contributions

TE conceived the study, gathered and reviewed literature, participated in the field work and manuscript drafting, AE participated in literature gathering and review, field work and manuscript drafting, AN and OP participated in the field work. All authors read and approved on the manuscript.

## Consent

Verbal informed consent was obtained from the participants of this study
